# Conservation and divergence of abiotic stress-responsive gene co-expression networks in diploid and tetraploid cotton

**DOI:** 10.3389/fpls.2026.1889274

**Published:** 2026-08-12

**Authors:** Heng Wang, Yumeng Zhu, Ting Zhao, Xueying Guan, Junduo Wang, Luyao Wang

**Affiliations:** 1 Zhejiang Provincial Key Laboratory of Crop Genetic Resources, Institute of Crop Science, Plant Precision Breeding Academy, College of Agriculture and Biotechnology, Zhejiang University, Hangzhou, China; 2 Xinjiang Key Laboratory of Cotton Genetic Improvement and Intelligent Production/Xinjiang Cotton Technology Innovation Center, Cotton Research Institute of Academy of Agricultural Sciences of Xinjiang Uyghur Autonomous Region (National Cotton Engineering Technology Research Center), Urumuqi, China; 3 Tropical Crops Genetic Resources Institute, Chinese Academy of Tropical Agricultural Sciences / Key Laboratory of Crop Gene Resources and Germplasm Enhancement in Southern China, Ministry of Agriculture and Rual Affairs / Key Laboratory of Tropical Crops Germplasm Resources Genetic Improvement and Innovation of Hainan Province, Haikou, China

**Keywords:** abiotic stress, comparative transcriptome, cotton, gene co-expression network, transploidy species comparison

## Abstract

Cotton (*Gossypium* spp.) is highly susceptible to abiotic stresses, including temperature extremes, drought, and salinity. The diploid species *Gossypium arboreum* retains stress adaptive traits that are poorly represented in modern tetraploid cultivars, yet the evolutionary and regulatory bases of these ploidy associated differences remain insufficiently understood. Here, we performed a comparative transcriptomic analysis of 54 seedling leaf RNA-seq libraries from *Gossypium arboreum*, *Gossypium hirsutum*, and *Gossypium barbadense* subjected to four abiotic stress treatments. By integrating standardized cross-species normalization with an optimized gene co-expression network framework, we minimized interspecific technical heterogeneity and enabled robust comparison of stress responsive regulatory architectures. Consensus network analysis revealed marked topological divergence between diploid and tetraploid cotton. The *G. arboreum* A_2_ network showed strong structural conservation with the A_t_ subgenome of *G. hirsutum*, whereas extensive co-expression rewiring was detected in the A_t_ subgenome of *G. barbadense*, which may reflect lineage-specific regulatory remodeling of stress-response programs after allopolyploidization. By integrating conserved multi-species networks with five genome or subgenome specific networks, we identified 54 core cold-repressed candidate genes, including a BBR-BPC transcription factor potentially associated with ethylene related stress signaling. The divergent topology of the cold-responsive subnetwork defined by these candidates suggests extensive transcriptional reprogramming associated with polyploid evolution. Module preservation analysis also uncovered an A_2_-specific heat-responsive module centered on an ERF transcription factor. In a distinct drought/heat co-responsive module, another ERF was predicted to coordinate both a heat shock factor (HSF) and an ABA responsive TALE transcription factor, suggesting potential regulatory crosstalk between heat and drought signaling pathways. Collectively, our study resolves conserved and lineage-specific components of cotton abiotic stress regulation and provides candidate regulators for resilience oriented molecular breeding.

## 1 Introduction

Cotton is the world’s foremost source of natural textile fiber and among the most economically significant cash crops globally. Over the past six millennia, four *Gossypium* species underwent independent domestication: the diploid A-genome species *Gossypium arboreum* (*G. arboreum*) and *Gossypium herbaceum* (*G. herbaceum*), and the allotetraploid AD-genome species *Gossypium hirsutum* (*G. hirsutum*) and *Gossypium barbadense* (*G. barbadense*), *G. arboreum* is hypothesized to have originated in Madagascar or the Indus Valley before expanding across Africa and Asia (Wendel et al., 2010). All A-genome species, including *G. arboreum*, likely diverged from a common ancestor through subsequent independent lineages (Huang et al., 2020). In contrast, *G. hirsutum* and *G. barbadense* share a monophyletic origin derived from a single allopolyploidization event between an ancestral A-genome progenitor and a D-genome relative resembling *Gossypium raimondii* (*G. raimondii*) that occurred approximately 1.0-1.5 million years ago (Endrizzi et al., 1985; Wendel, 1989; Huang et al., 2020; Zhang et al., 2015). Following polyploid formation, these two tetraploid species underwent parallel yet distinct domestication trajectories. Currently, *G. hirsutum* and *G. barbadense* dominate the global cotton industry, collectively accounting for over 95% of total fiber production.

Exacerbated by intensifying global climate change, cotton cultivation faces escalating threats from multifaceted environmental constraints. A substantial body of literature has elucidated the molecular mechanisms underlying abiotic stress tolerance in cotton, encompassing responses to cold (Wang et al., 2023), heat (Dev et al., 2024), drought (Ullah et al., 2017), and salinity (Zhang et al., 2021). Concurrently, pivotal transcription factor families, most notably WRKY, MYB, ERF and NF-YA, have been identified as central hubs within intricate regulatory networks, largely orchestrated by phytohormone signaling cascades including abscisic acid (ABA), ethylene, and Methyl jasmonate (MeJA) (Butt et al., 2017; Luo et al., 2020; Zhang et al., 2020; Lu et al., 2021).

Despite these advances, prior investigations have predominantly focused on the widely cultivated *G. hirsutum* and the superior-quality yet stress-vulnerable *G. barbadense*, leaving diploid progenitors comparatively neglected. Diploid species, such as *G. arboreum*, did not undergo the radical chromosomal rearrangements and whole-genome duplication characteristic of allotetraploids, thereby potentially preserving elite alleles that govern both stress resilience (Kantartzi et al., 2009; Gogari and Radha, 2025) and fiber quality (Lee and Fang, 2015). While the genetic potential of *G. arboreum* for the improvement of tetraploid cultivars has been substantiated (Chen et al., 2015; Feng et al., 2021; Sun et al., 2024), deciphering the adaptive divergence between diploid and polyploid cottons, as well as the evolutionary trajectories of stress resistance modulated by allopolyploidization, remains a fundamental yet unresolved inquiry in cotton evolutionary biology and resilience-targeted breeding.

Theoretically, polyploidy often confers broader environmental plasticity, a phenomenon largely attributed to genomic redundancy and duplicated gene dosage. A hallmark example is the tetraploid *Centaurea stoebe*, which exhibits broader ecological tolerance than its diploid counterparts, thereby facilitating its invasive success in novel habitats (Hahn et al., 2012). As a classical model for studying allopolyploidization, *Gossypium* has been intensively investigated for the impact of genome doubling on stress resilience and developmental plasticity. Our previous research established that allopolyploidization precipitated the emergence of numerous novel non-coding RNAs (ncRNAs), several of which orchestrate development and abiotic stress responses in cotton (Zhao et al., 2018; Wang et al., 2021). Notably, the long intergenic non-coding RNA (lincRNA) *DAN1*, inherited from the ancestral diploid A-genome, trans-regulates drought-responsive gene expression (Tao et al., 2021); conversely, silencing the lincRNA *XH123* significantly heightens sensitivity to cold stress in cotton seedlings (Cao et al., 2021). However, recent findings have revealed a nuanced complexity introduced by allopolyploidization that challenges the conventional “polyploid superiority” paradigm. For instance, comparative assessments of salinity tolerance revealed a hierarchy of A-genome > AD-genome > D-genome, yet with substantial intragenomic variation, indicating that allotetraploid cotton does not universally manifest superior salinity tolerance relative to its diploid progenitors (Dong et al., 2020). Such unpredictable evolutionary dynamics highlight the need to comprehensively dissect the divergence in molecular regulatory architectures between diploid and allotetraploid cottons under abiotic stresses.

Transcriptional regulation in allopolyploid cotton is characterized by pronounced non-additive effects, with homoeologous gene pairs frequently exhibiting biased expression toward either subgenome (Grover et al., 2012). Accumulating evidence indicates that across diverse tissues and developmental stages in *G. hirsutum*, approximately 20% to 55% of homoeologs display significant expression bias toward either the A_t_ or D_t_ subgenome (Yoo and Wendel, 2014; Hovav et al., 2015; Zhang et al., 2015). This regulatory complexity, while a defining feature of allopolyploid evolution, poses a formidable challenge for comparative transcriptomic analyses across disparate ploidy levels. Constrained by historical genomic resources, early investigations predominantly utilized a single reference, typically the *G. raimondii* (D_5_) genome, as a unified template for cross-ploidy comparisons (Hu et al., 2016). However, following the release of high-quality reference genomes for *G. hirsutum*, *G. barbadense*, and *G. arboreum* (Zhang et al., 2015; Hu et al., 2019; Huang et al., 2020), this strategy fails to meet current standards for precise quantification, as it cannot mitigate alignment biases inherent to genomic heterogeneity. Conversely, although employing species-specific reference genomes circumvents such biases, it introduces a critical methodological bottleneck: the difficulty in distinguishing genuine evolutionary divergence from confounding technical variations.

By leveraging high-resolution transcriptomic datasets from *G. arboreum*, *G. hirsutum*, and *G. barbadense* under four representative abiotic stresses, we developed an integrated standardization framework that enables direct comparison of orthologous and homoeologous gene expression across diploid genomes and allotetraploid subgenomes. Building upon this framework, we integrated multi-species and genome-specific co-expression network analyses to characterize both conserved and lineage-specific stress-responsive regulatory architectures. These analyses provide new insights into the evolutionary conservation and divergence of abiotic stress-responsive transcriptional networks while prioritizing candidate regulatory genes and network modules associated with stress adaptation. Together, this work provides a comparative framework for investigating regulatory network evolution during cotton polyploidization and offers methodological guidance and candidate genes for future functional studies and resilience-oriented cotton improvement.

## 2 Materials and methods

### 2.1 Plant materials

The three cotton accessions used in this research were *Gossypium arboreum* (A_2_), accession SXY1; *Gossypium hirsutum* (AD)_1_, accession Texas Marker-1 (TM1); *Gossypium barbadense* (AD)_2_, accession H7124. Plants were cultivated in a growth chamber at 25 to 28°C with a light/dark cycle of 16/8 hours. All were planted in 6.5 cm * 6.5 cm plastic pots filled with a 1:1 (v/v) mixture of commercial humus: commercial vermiculite.

### 2.2 Stress treatment and chlorophyll content

For cold stress (CS) treatment and heat stress (HS) treatment, three-leaf stage seedlings were transferred to a 4 °C chamber grown for 48 h or a 42 °C chamber grown for 72 h, in order to observe phenotypic differences, and the second fully expanded leaves from the apex were subsequently harvested for chlorophyll quantification. For drought stress (DS) and salinity stress (SS) treatments, seedlings at three-leaf stage were subjected with water withholding or irrigated with150 mM NaCl solution for 15 days. The second fully expanded leaf from the apex was then harvested for chlorophyll content determination. To determine chlorophyll content, 100 mg of fresh leaf material was cut into small segments and immersed in 5 mL of 80% acetone (v/v) for 48 h in the dark. The absorbance of the supernatant was determined spectrophotometrically using a Multi-Detection Microplate Reader. The concentrations of chlorophyll a (Chla), chlorophyll b (Chlb), total chlorophyll (Chla+b) and the ratio of chlorophyll a to b (Chla/b) were calculated according to the method described by (Porra et al., 1989).

### 2.3 Genome resources and annotations

Three reference cotton genomes used for data analysis were *G. arboreum* genome (WHU_SXY1_V1) (Huang et al., 2020), *G. hirsutum* genome (ZJU_TM1_V2.1) (Hu et al., 2019) and *G.barbadense* genome (ZJU_H7124_V1.1) (Hu et al., 2019). The cotton reference genome and annotation data sets, including transcription factors, Gene Ontology (GO) annotation, were retrieved from COTTONOMICS (http://cotton.zju.edu.cn/), and CottonGen (https://www.cottongen.org/) (Yu et al., 2026). The *Arabidopsis thaliana* genome annotation was retrieved from The Arabidopsis Information Resource (TAIR) (https://www.arabidopsis.org/) (Reiser et al., 2024). One-to-one orthologous and homoeologous genes were identified using an established pipeline based on BLAST (v2.13.0) software and OrthoMCL (v2.0.9) software (Zhang et al., 2025a). Briefly, all-versus-all protein sequence comparisons were performed using BLAST (v2.13.0) (Altschul et al., 1997) with an e-value threshold of 10^-3^, followed by normalization of bit scores to account for gene length and phylogenetic distance. Reciprocal Best Length-Normalized Hits (RBNHs) were subsequently used to define sequence similarity thresholds for putative orthologs. An orthology graph was then constructed from normalized sequence similarities and clustered using OrthoMCL (v2.0.9) (Li et al., 2003) to identify one-to-one orthologous groups across the three cotton species and *A. thaliana.*

### 2.4 RNA extraction and transcriptome sequencing

Cotton seedlings with three expanded leaves were used for RNA-seq with stress treatments. CS treatment was carried out at 4 °C, HS at 42 °C, SS with 150 mM NaCl solution. For RNA-seq analysis, DS was imposed by exposing seedlings to air-drying conditions to rapidly induce dehydration stress. Seedlings were carefully removed from the original substrate with intact root systems, residual substrate was gently removed, and roots were briefly rinsed with water before air-drying exposure. CS was performed as an independent experiment, with samples of leaf tissues harvested after 48 h together with the corresponding control group (CK). HS, DS, and SS were conducted in a separate experimental batch, and samples of leaf tissues were harvested after 6 h together with an independent time-matched control group (CK_2). For all downstream analyses, each stress treatment was compared exclusively with its corresponding control collected under identical sampling conditions. All harvested leaf tissues were frozen with liquid nitrogen for RNA extraction and sequencing with three biological replicates per group.

RNA sequencing libraries were constructed using the NEBNext Ultra II RNA Library Prep Kit for Illumina (NEB, E7770S) and sequenced on the Illumina NovaSeq 6000 platform to generate 150-bp paired-end reads at BerryGenomics Company (http://www.berrygenomics.com/, Beijing, China). Raw sequencing reads were processed using fastp (v0.12.2) with default parameters for quality control (Chen et al., 2018). Clean reads were aligned to the reference genome using HISAT2 (v2.1.0) (Pertea et al., 2016), and the resulting BAM files were sorted and indexed using SAMtools (v1.10) (Li et al., 2009). Transcript assembly and gene expression quantification, including fragments per kilobase of transcript per million mapped reads (FPKM) and transcripts per million (TPM), were calculated by StringTie (v2.1.4) with default parameters (Pertea et al., 2015). Raw read counts were calculated by htseq-count script of HTSeq (v0.12.4) with the parameters “-f bam -s no” (Anders et al., 2015). The Differentially expressed genes (DEGs) were determined using DESeq2 (v1.48.0) (Love et al., 2014) with |log_2_(fold-change)| ≥ 1 and a false discovery rate (FDR) < 0.05.

### 2.5 Expression data normalization

TPM values of one-to-one orthologous and homoeologous genes from the five genomic datasets (*G. arboreum* SXY1_A_2_, *G. hirsutum* TM1_A_t_/D_t_, *G. barbadense* H7124_A_t_/D_t_) were integrated into a unified expression matrix. Low-expression genes were filtered out, retaining only gene rows with TPM values greater than 0.5 in at least 3 samples. The filtered matrix was subsequently subjected to log_2_ transformation followed by quantile normalization, yielding the first normalized expression matrix (Normalized Expression 1, NE1). Then, batch effects attributable to genomic background were corrected using the removeBatchEffect function implemented in the R package limma (v3.64.0) (Ritchie et al., 2015), with the five genome identifiers specified as the batch factor while preserving the variation attributable to stress treatments using the experimental design matrix, generating the second normalized expression matrix (Normalized Expression 2, NE2). Principal component analysis (PCA) was performed with the R package FactoMineR (v2.11) (Lê et al., 2008) to assess the effectiveness of data normalization and batch-effect correction.

### 2.6 Multi-species conservative gene co-expression network analysis

Here, we performed bootstrapping re-sampling co-clustering network analysis (BRSCCNA) using the NE2 matrix to identify conserved multi-species gene co-expression network (Shahan et al., 2018). (BRSCCNA, also known as co-clustering network analysis, CCNA, was named here to distinguish it from Consensus Network Analysis in weighted gene co-expression network analysis). Firstly, three key parameters, soft threshold {1, 2, 4, 8, 12, 14, 16, 18}, minimum module size {30, 60, 90, 120, 150, 180, 210}, and whether to merge similar expression modules [True/False], were randomly sampled with replacement 1000 times, and meanwhile 80% of the genes were randomly selected with randomly sampled three key parameters each time for a individual standard weighted gene co-expression network analysis (WGCNA) (Langfelder and Horvath, 2008). After 1000 standard WGCNA network construction, the number of times of each gene simultaneously selected with other genes was counted as matrix *A*, and the number of times each gene be divided into the same module with other genes was counted as matrix *B*. Matrix *C*, calculated by matrix *B*/*A*, was used to replace the adjacency matrix in the standard WGCNA. For BRSCCNA, the dynamic tree cut parameter was set to minClusterSize = 30, and module merging was performed using cutHeight = 0.25. The module eigengene (ME), defined as the first principal component of the scaled module expression profiles, was used to characterize the gene expression pattern for a given module. The measure of intramodular connectivity is mathematically equivalent to the module membership of a gene, MM or kME, which was estimated by the Pearson correlation between the expression level of that gene and the module eigengene (Horvath and Dong, 2008). For module-trait correlation analysis, each treatment group, including the four stress treatments together with CK and CK_2, was treated as an independent binary trait. Samples belonging to the corresponding treatment group were assigned a value of 1, whereas all remaining samples were assigned a value of 0. Pearson correlations were performed between module eigengenes and these binary traits to identify stress- associated modules. Significant module-stress associations were considered when Benjamini-Hochberg (BH) adjusted *P*-values (*q*-value) were < 0.05. The correlations between individual gene expression profiles and stress were defined as Gene Significance (GS). In CS-related modules, module hub genes were identified when kME > 0.95 due to huge module size. In other modules, module hub genes were identified when kME > 0.9.

### 2.7 Consensus analysis and module preservation

We constructed 5 genome networks individually by standard WGCNA from SXY1_A_2_ (SA), TM1_A_t_ (TA), TM1_D_t_ (TD), H7124_A_t_ (HA) and H7124_D_t_ (HD) submatrices of NE1 matrix. Consensus networks were constructed using the minimum topological overlap similarity measure (TOM) derived from individually constructed and calibrated networks. Genome-specific WGCNA and consensus network analyses were conducted using signed networks (networkType = “signed”), with corType = “pearson”, TOMType = “signed”, minModuleSize = 30 and mergeCutHeight = 0.25. The soft-thresholding powers were 18, 16, 16, 16, and 14 for SA, TA, TD, HA, and HD, respectively. Two complementary statistics were used to evaluate network preservation at different organizational levels. The density *D* of the eigengene network was defined as an aggregated measure of adjacency preservation of module eigengene, which measures the preservation of global eigengene connectivity between two co-expression networks and therefore reflects the similarity of overall network architecture (Langfelder and Horvath, 2007). The *D* was calculated by setCorrelationPreservation function in R package WGCNA (v1.73) with default parameters. The closer the *D* is to 1, the higher conservative the two eigengene networks are. To test for differences between two eigengene preservation, paired Student’s *t*-test was applied for mean connectivity of the adjacency preservation. Additionally, the module preservation statistic *Z_summary_* evaluates the preservation of the internal topology of individual modules by integrating multiple density- and connectivity-based preservation measures (Langfelder et al., 2011). Module preservation of different networks was performed by the R package WGCNA (v1.73) function modulePreservation with *Z_summary_* measure through 100 permutations (networkType = “signed”). In general, modules with *Z_summary_* > 10 are interpreted as strongly preserved, whereas those with *Z_summary_* between 2 and 10 are weakly to moderately preserved, and those with *Z_summary_* < 2 exhibit no evidence of preservation. Because global network architecture and individual module topology represent different hierarchical properties of co-expression networks, both *D* and *Z_summary_* were used to provide complementary assessments of network preservation.

### 2.8 GO enrichment analysis

We conducted the gene ontology (GO) enrichment analysis by the funcion enricher in the R package clusterProfiler (v4.16.0) (Wu et al., 2021). The GO terms exhibiting a BH adjusted *P*-value of < 0.05 were significantly enriched.

### 2.9 Protein-protein interaction analysis

Based on orthologous genes of *Arabidopsis thaliana*, we conducted protein-protein interaction analysis by STRING database (v12.0) (Szklarczyk et al., 2022) with interaction score > 0.4 and an FDR cutoff of 0.05.

### 2.10 Predicted regulatory network of transcription factors

Transcription factors binding motifs of orthologous genes in *Arabidopsis thaliana*, obtained from PlantTFDB (v5.0) (https://planttfdb.gao-lab.org/) (Jin et al., 2017), were used as TF binding motifs in cotton network modules. We scanned for matches between all genes’ promoters, ranging from 2,000 bp upstream to 500 bp downstream, and TFs in the given module, performing by FIMO (v5.5.7) (Grant et al., 2011) with a match *P*-value threshold of 1 × 10⁻^4^.

### 2.11 Statistical analysis and plots

Multiple group comparisons for the chlorophyll content data were performed using the R package dunn.test (v1.3.6) (https://CRAN.R-project.org/package=dunn.test) with the false discovery rate method “bh” as a *post hoc* Dunn’s test after application of the Kruskal-Wallis test using kruskal.test function in R version 4.5.0. Heatmaps were generated in R (v4.5.0) using the pheatmap package (v1.0.12) and the labeledHeatmap function implemented in the WGCNA package (v1.73). Dot plots were generated in R (v4.5.0) using the dotplot function from the clusterProfiler package (v4.16.0). Network visualizations were constructed using Cytoscape (v3.10.3). All other statistical graphics were generated in R (v4.5.0) using the ggplot2 package (v3.5.2).

## 3 Results

### 3.1 Phenotypic responses to abiotic stresses across cotton species

**Figure 1:**
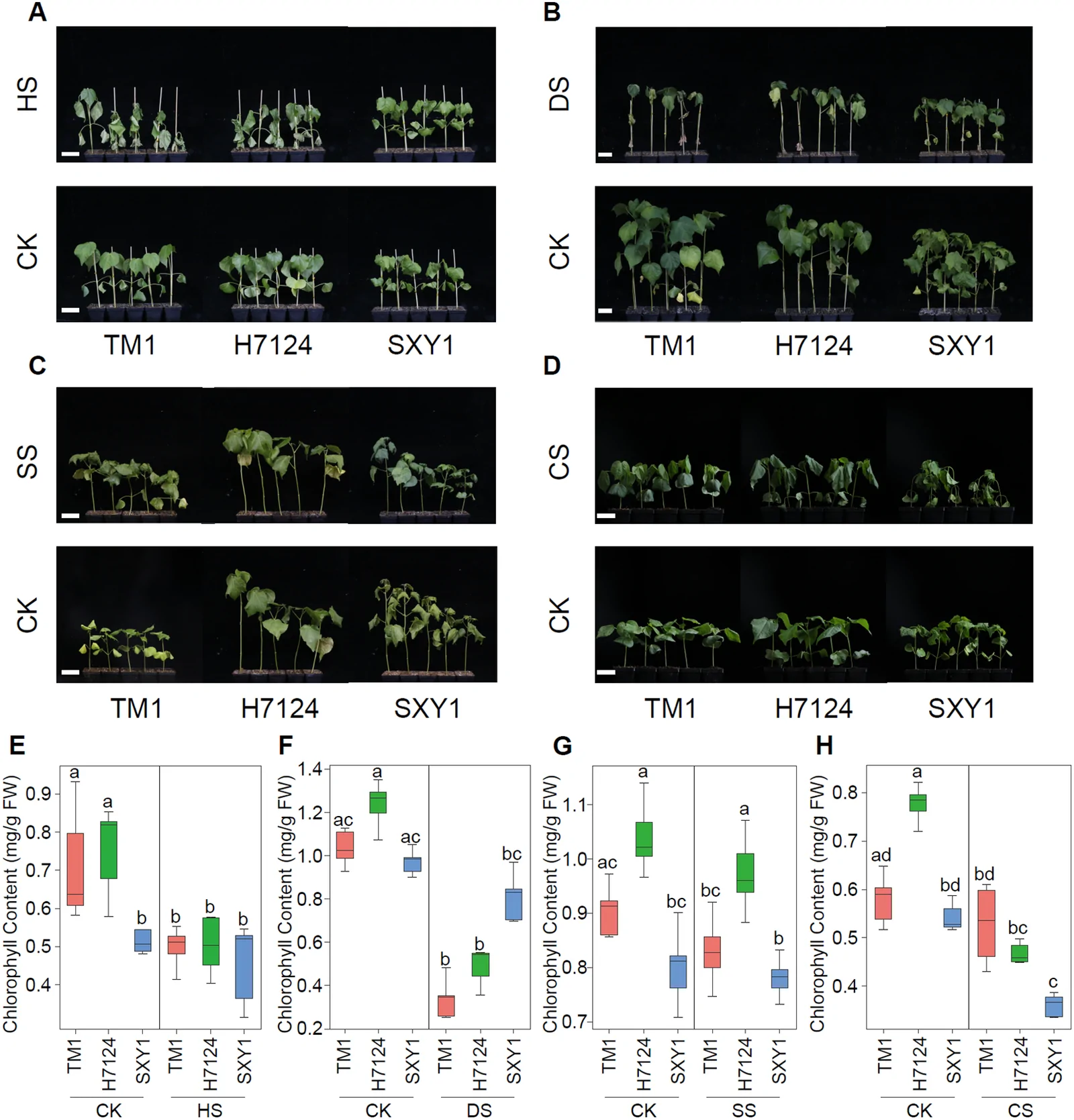
Comparative analysis of abiotic stress resilience and chlorophyll dynamics among three cotton species. *Gossypium hirsutum* (TM1), *Gossypium barbadense* (H7124), and *Gossypium arboreum* (SXY1) were evaluated at the seedling stage. (**A–D)** Phenotypic manifestations of TM1, H7124, and SXY1 under control conditions and distinct abiotic stressors: heat stress (HS, 42 °C for 72 h), drought stress (DS, water withholding for 15 d), salinity stress (SS, 150 mM NaCl for 15 d), and cold stress (CS, 4 °C for 48 h). Scale bar: 6.5 cm. **(E–H)** Comparative assessment of total leaf chlorophyll content across the three species under above four stresses (n = 5 independent plants per treatment group). Statistical significance is indicated by distinct lowercase letters (Kruskal-Wallis test followed by *post hoc* Dunn’s test with Benjamini-Hochberg adjusted *P* < 0.05).

To evaluate the phenotypic plasticity across different cotton species, we subjected *G. hirsutum* (TM1), *G. barbadense* (H7124), and *G. arboreum* (SXY1) to various seedling-stage abiotic stressors. The SXY1 demonstrated robust performance under heat (42 °C for 72 h) and prolonged drought (15 days) conditions; specifically, SXY1 maintained leaf turgor and erect growth (**Figures 1A, B**), with chlorophyll concentrations comparable to control levels (**Figures 1E, F**). Under salinity stress (150 mM NaCl for 15 days), no significant differences in growth vigor or chlorophyll content were observed among the three species (**Figures 1C, G**). This is consistent with the notion that *Gossypium* species are inherently moderately salt-tolerant. Under cold stress (4 °C for 48 h), both H7124 and SXY1 showed more severe injury symptoms (**Figure 1D**), including severe leaf wilting, chlorosis, and marginal curling. These phenotypic changes were consistent with a significant reduction in chlorophyll content relative to their corresponding controls (**Figure 1H**). In contrast, TM1 displayed only mild wilting without significant alteration in chlorophyll levels (**Figure 1H**), suggesting that TM1 possesses superior cold tolerance compared with H7124 and SXY1.

### 3.2 Transcriptomic landscape under abiotic stresses

**Figure 2:**
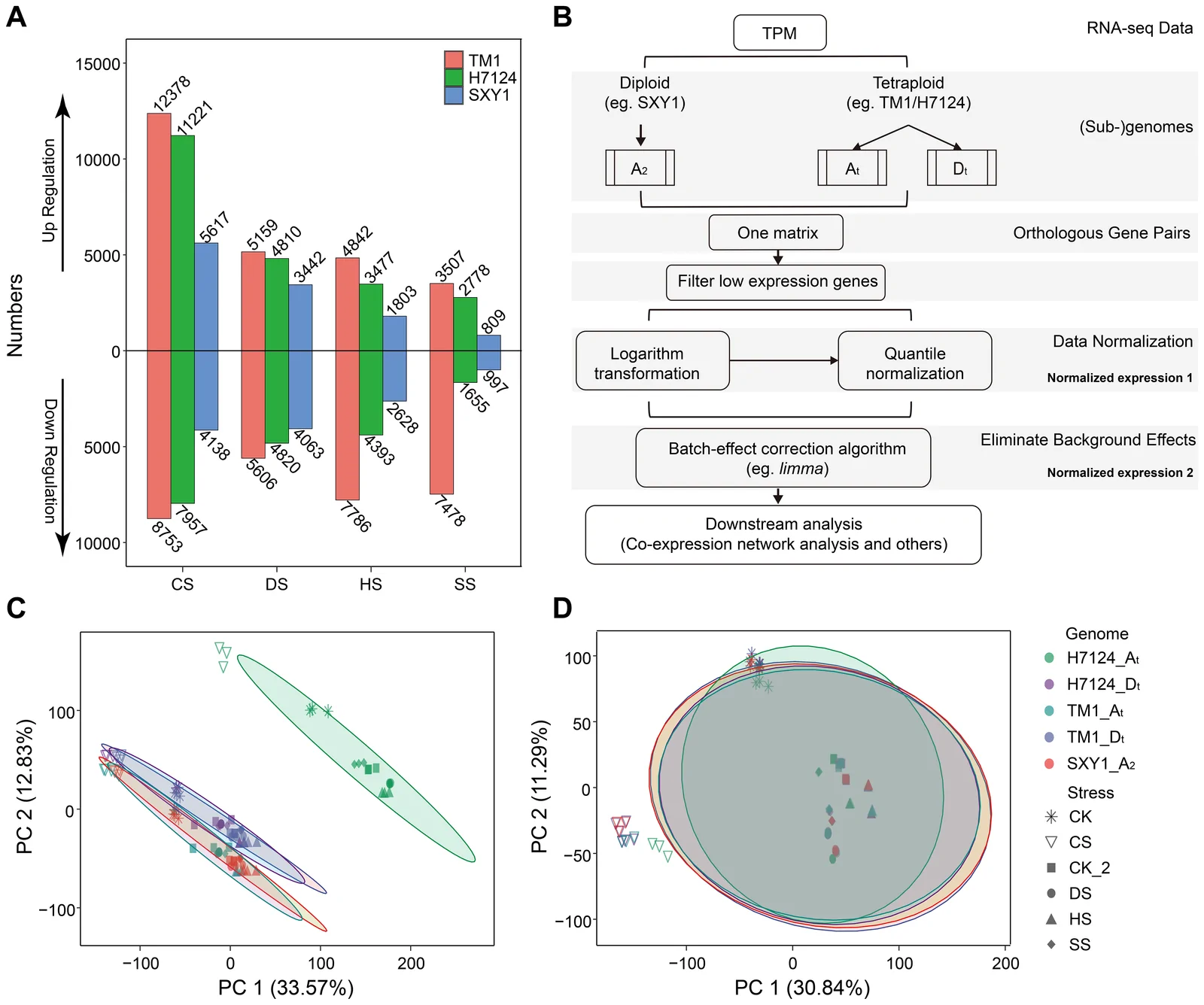
Identification of differentially expressed genes (DEGs) and implementation of the data-standardization pipeline. **(A)** Quantification of DEGs between control and stress-treated cohorts for TM1, H7124, and SXY1. Upregulated and downregulated gene counts are delineated above and below the horizontal axis, respectively. **(B)** Schematic workflow of the cross-ploidy transcriptomic normalization strategy. **(C, D)** Principal Component Analysis (PCA) of transcript expression profiles prior to **(C)** and following **(D)** batch-effect correction. Point colors designate the subgenomes of TM1, H7124, and SXY1, while point shapes represent distinct treatment groups. CK denotes the control group for cold stress (CS), whereas CK_2 serves as the common control for heat stress (HS), drought stress(DS), and salinity stress (SS).

To characterize early transcriptional reprogramming under abiotic stresses, heat-, drought-, and salinity-treated leaf samples were harvested at 6 h post-treatment, whereas cold-treated samples were collected after 48 h in an independent experiment using a matched control group. All samples were subjected to paired-end RNA sequencing (PE150) with three biological replicates. Given that abiotic stresses trigger rapid transcriptomic reprogramming within hours, preceding extensive secondary and pleiotropic responses (Hu et al., 2019), these sampling time points were chosen to capture primary stress-responsive transcriptional changes. In contrast, phenotypic assessments and chlorophyll measurements were conducted after prolonged stress exposure to evaluate integrated physiological responses and interspecific variation in stress tolerance. Together, these analyses characterize distinct temporal phases of the stress response, providing complementary perspectives on early molecular responses and later physiological outcomes. A total of 1.53 billion reads were generated across 54 quality-controlled libraries, with GC content ranging from 43% to 46% (**Supplementary Table 1**). Of these, approximately 1.47 billion (96.10%) were successfully aligned to their respective reference genomes, among which 1.34 billion (87.42%) were uniquely mapped (**Supplementary Table 1**). Furthermore, the Pearson correlation coefficients of TPM values between biological replicates ranged from 0.98 to 1.00 (**Supplementary Figure 1**), indicating high reproducibility. Differential expression analysis showed that TM1 exhibited the largest number of differentially expressed genes (DEGs) among the three cotton species under all four abiotic stress conditions (**Figure 2A**).

### 3.3 Standardization pipeline for cross-ploidy comparative transcriptomics

Integrating transcriptomic datasets across varying ploidy levels presents formidable biological and bioinformatic challenges. Differences in ploidy not only alter the physical scale of genomes but also drive homoeologous expression bias (Grover et al., 2012). At the bioinformatic level, heterogeneities in reference genome strategies frequently introduce systematic errors, rendering quantitative data non-comparable across subgenomes and species. To circumvent these limitations, we developed a robust data standardization pipeline (**Figure 2B**). We first integrated the expression profiles of one-to-one orthologous and homoeologous genes from the diploid A genome and the A_t_ and D_t_ subgenomes of the allotetraploids into a unified expression matrix. Following log-transformation and quantile normalization, we generated the Normalized Expression 1 (NE1) matrix, which minimized distributional discrepancies and stabilized expression variance across genomes (**Figure 2B****;** **Supplementary Figure 2**). Although PCA of NE1 revealed clear segregation among stress treatments, substantial genome-dependent clustering persisted within individual treatment groups, indicating that genomic background contributed substantial systematic variation to the global transcriptome (**Figure 2C**). To minimize genome-associated effects and facilitate conserved comparative network analyses, we further corrected the NE1 matrix using the batch effect adjustment algorithm implemented in the R package limma (Ritchie et al., 2015), treating genome identity as the batch factor while retaining stress-associated biological variation. The resulting Normalized Expression 2 (NE2) matrix effectively reduced genome-driven systematic bias, as demonstrated by PCA, in which sample variation was predominantly explained by stress treatments rather than genomic background (**Figure 2D**). Collectively, this study establishes a rigorous cross-ploidy and cross-species transcriptomic comparative pipeline, providing a reliable technical framework for deciphering the molecular evolutionary mechanisms underlying stress resilience during polyploidization.

### 3.4 Multi-species conserved co-expression networks

Gene co-expression networks consist of highly interconnected gene clusters, and topological analyses provide insights into the regulatory mechanisms governing complex biological processes, such as abiotic stress responses. In allotetraploid cotton, genomic redundancy profoundly confounds homoeologous functional divergence and transcriptional regulation. Deconstructing tetraploid genomes into subgenomes and integrating them with their diploid counterparts offers a powerful approach to systematically dissect the topological conservation and evolutionary divergence of gene regulatory networks across different genomic lineages.

The standard WGCNA framework relies heavily on the parameterization of soft-thresholding powers and minimum module sizes, where minor changes in these parameters frequently cause substantial changes in module partitioning. To enhance the robustness of network inference, this study employed an optimized pipeline incorporating iterative re-sampling and parametric bootstrapping, termed Bootstrapping Re-sampling Co-clustering Network Analysis (BRSCCNA). This workflow executes *N* (*N* = 1000) bootstrap sampling events with replacement across a candidate space of soft-thresholding powers and other parameters, while randomly subsetting 80% of genes in each iteration. By integrating clustering outcomes across these simulations, a consensus probability matrix was constructed, thereby mitigating parameter selection bias and substantially enhancing network structural stability.

**Figure 3:**
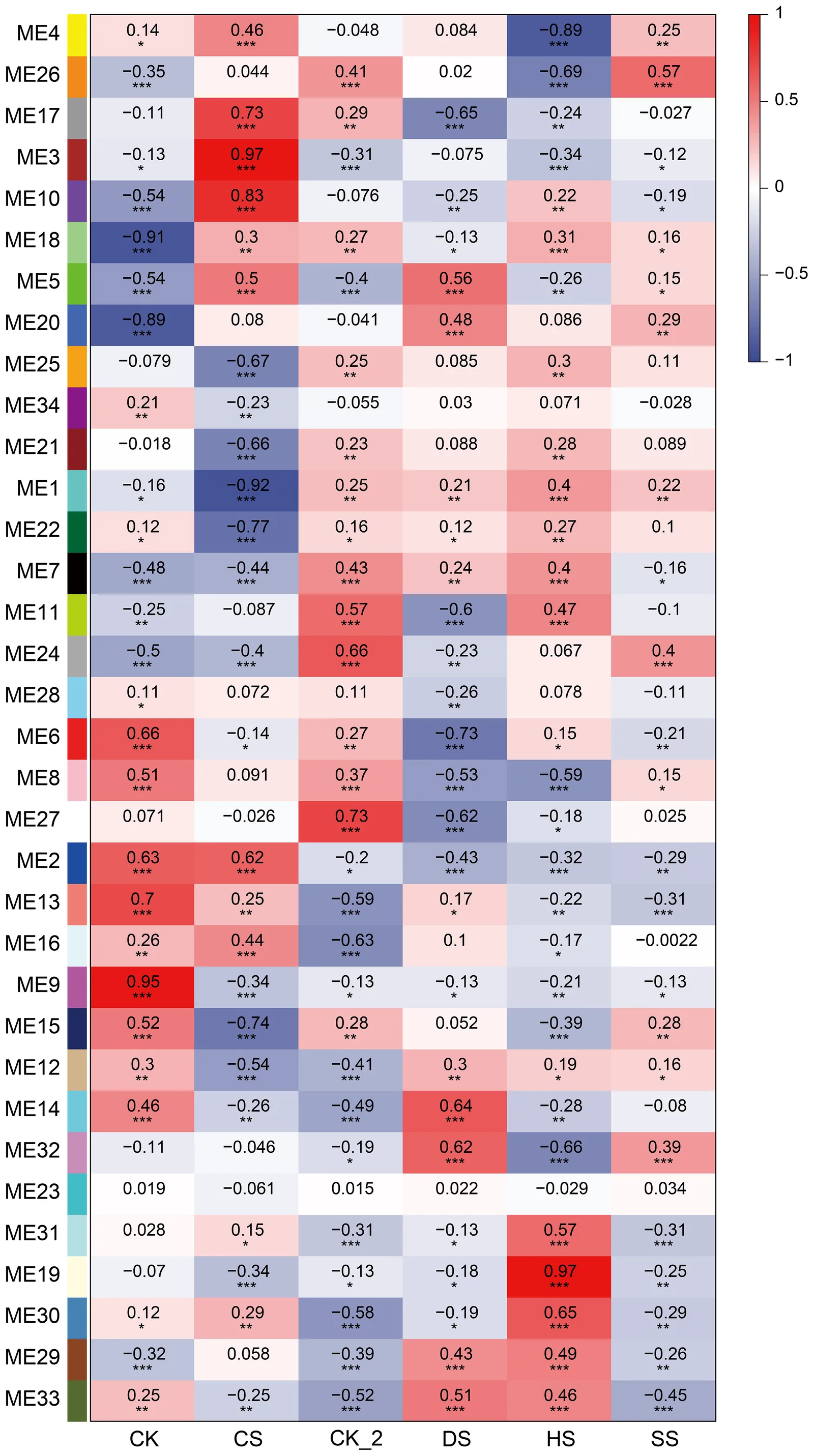
Correlations between multi-species conserved co-expression modules and abiotic stressors. Module eigengenes (rows, designated by module ID and assigned color) were correlated with specific abiotic stress cohorts (columns). CK denotes the control group associated with cold stress (CS), while CK_2 serves as the unified control for drought stress (DS), heat stress (HS), and salinity stress (SS). The heatmap color scale represents Pearson correlation coefficients, with asterisks indicating Benjamini-Hochberg (BH) adjusted *P*-values (*P < 0.05; **P <0.01; ***P <0.001).

Utilizing the standardized NE2 matrix of 18,335 orthologous/homoeologous gene sets, BRSCCNA identified 34 robust co-expression modules (M1-M34, **Figure 3**), with sizes ranging from 31 to 7,051 genes. The module eigengene, defined as the first principal component, serves as a proxy for the collective expression pattern of its respective module. By correlating module eigengenes with specific stress treatments, complemented by linear regression between Gene Significance (GS) and Module Membership (kME) (**Supplementary Figures 3**, **4**), we prioritized key stress-responsive modules. Notably, modules M3, M19, and M14/M32 exhibited strong positive correlations with cold, heat, and drought, respectively, whereas M1, M4, and M6 displayed pronounced negative correlations. Consistent with the uniform physiological resilience to salinity observed phenotypically, the global transcriptional network exhibited decentralized response profiles, among which module M26 emerged as the most prominent representative capturing the conserved salinity-responsive circuitry. Cross-species eigengene profile analysis revealed that among the drought-associated modules (M14, M32, and M6), the absolute expression amplitudes of SXY1_A_2_ were comparable to or slightly higher than those of H7124_A_t_/D_t_, while remaining significantly higher than those of TM1_A_t_/D_t_ (**Supplementary Figure 5**). This transcriptional prevalence potentially underpins the robust drought tolerance of diploid cotton.

### 3.5 Stress-responsive candidate genes from multi-species co-expression networks

By prioritizing genes with high module membership (kME) within significantly correlated modules, we identified a set of hub genes potentially regulating the abiotic stress response (**Supplementary Table 2**). Within the cold-responsive module M3, 34 hub genes were identified, spanning key transcription factor (TF) families including GRAS, NF-X1, and C2H2. *Arabidopsis thaliana* orthologous annotation suggests that a specific GRAS TF (*OG0005416*) interacts with TGA2 to modulate the transcription of stress-responsive genes, whereas a C2H2 TF (*OG0024646*) is likely involved in the regulation of stress-associated DNA transcription. Furthermore, module M3 exhibited significant Gene Ontology (GO) enrichment in protein ubiquitination pathways (**Supplementary Figure 7A**), encompassing ubiquitin ligase and transferase activities. Given the pivotal roles of ubiquitination in programmed cell death and stress adaptation (Chen et al., 2019), these results suggest that protein ubiquitination may contribute to cold-stress adaptation through targeted protein degradation. In contrast, the cold-repressed module M1 contained 59 hub genes, notably featuring BBR-BPC, ZF-HD, and MYB-related TFs. The BBR-BPC TF (*OG0004882*), encoding a BPC6 ortholog, is predicted to participate in ethylene signaling. Accumulating evidence indicates that ethylene is an important regulator of plant cold-stress responses. Interestingly, the effects of ethylene on cold tolerance appear to be species- and context-dependent, with both positive and negative regulatory roles reported in upland cotton (*Gossypium hirsutum*) (Dong et al., 2025), rice (*Oryza sativa*) (Huang et al., 2025), tomato (*Solanum lycopersicum*) (Wang et al., 2024), apple (*Malus domestica*) (Zhang et al., 2025b), and trifoliate orange (*Poncirus trifoliata*) (Zhang et al., 2022b). These observations suggest that ethylene-mediated signaling may contribute to low-temperature responses in cotton, and BBR-BPC TF may represent as a promising candidate for further investigation of the regulatory networks associated with ethylene-mediated cold responses in cotton.

Analysis of heat stress responses revealed 6 and 15 hub genes in the positive module M19 and negative module M4, respectively (**Supplementary Table 2**). Key regulatory targets included a Class I small heat-shock protein (sHSP) gene (*OG0016447*) and multiple chaperones (e.g., HSP70 and HSP90 co-chaperones), such as *OG0018119*, *OG0020282*, and *OG0018702*. These findings underscore the central role of the HSP-chaperone interaction network in conferring heat tolerance. Within module M4, a hub MYB (*OG0020045*) known to regulate dehydration-responsive genes was identified, potentially mitigating heat-induced secondary water deficit.

In the drought-positive module M14, the presence of the ABA-mediated dehydration-responsive gene *RD22* (*RESPONSIVE TO DESICCATION 22*) (*OG0009672*) is consistent with a prominent contribution of ABA signaling to drought responsive transcriptional regulation in cotton. Module M32 contained a WRKY family transcription factor as a hub gene, further supporting the conserved association of WRKY proteins with plant stress-responsive regulatory networks (Yang et al., 2025). Conversely, hub genes in the drought-repressed module M6 were almost predominantly associated with photosystem components and photosynthetic regulators, with GO analysis showing pronounced enrichment in photosynthesis-related terms (**Supplementary Figure 6C**). This suggests that coordinated transcriptional repression of photosynthesis-associated genes may represent an adaptive response to water deficit, potentially contributing to the maintenance of physiological homeostasis and resource reallocation under drought conditions. Furthermore, in the salinity-positive module M26, hub genes involved in ethylene and jasmonic acid mediated signaling were identified, suggesting that hormonal crosstalk may participate in the regulatory network underlying cotton responses to salinity stress.

### 3.6 Consensus network analysis reveals pronounced interspecific divergence in stress-responsive architectures

In addition to the aforementioned methodologies, Consensus Analysis within the WGCNA framework represents another prevalent strategy for constructing conserved networks across multiple datasets. This approach integrates independent networks by calculating the minimum topological overlap measure (Minimum TOM) across individual datasets. In this study, we reconstructed independent stress responsive co-expression networks for each of the five genomes datasets, resulting in a consensus network of 32 consensus modules (consM1-consM32, cM1-cM32). To rigorously identify stress-associated consensus modules, we implemented stringent criteria: modules were retained only if they exhibited consistent correlation polarities across all five genomes and reached statistical significance (False Discovery Rate, FDR, BH adjusted *P* < 0.05) in at least one genome (**Supplementary Figure 7**). Overlap analysis confirmed that these significantly associated consensus modules were highly congruent with the multi-species conserved network modules established previously (**Supplementary Figure 8**), thereby cross-validating the reliability of our conserved network architecture.

**Figure 4:**
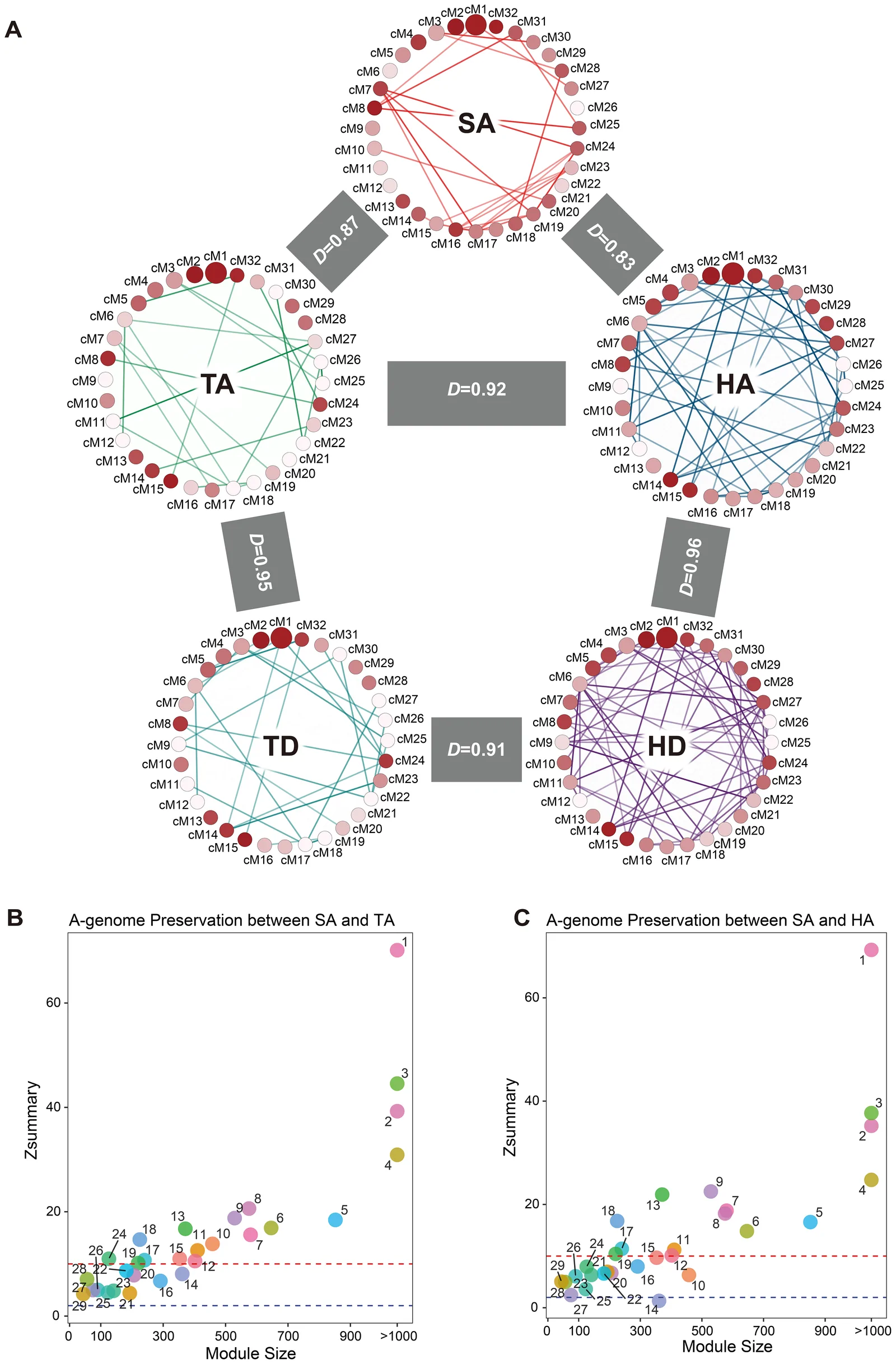
Topological preservation of consensus eigengene networks and A-genome networks across *G. arboreum*, *G. hirsutum* and *G. barbadense*. **(A)** Inter-module connectivity architecture. Consensus modules (nodes) are interconnected by edges if their eigengenes exhibit high transcriptional coordination (Pearson’s correlation *r* > 0.9) across all five genomes networks: *G. arboreum* (SXY1_A_2_, SA), *G. hirsutum* (TM1_A_t_, TA), (TM1_D_t_, TD), and *G. barbadense* (H7124_A_t_, HA), (H7124_D_t_, HD). Modules are arranged counterclockwise according to their identifiers. Node color intensity denotes the absolute correlation between module eigengenes and abiotic stressors, while node size is proportional to the gene membership of each consensus module. The preservation statistic *D* is provided for pairwise eigengene network comparisons, where values approaching 1.0 indicate heightened structural conservation. **(B, C)** Preservation of SXY1_A2 (SA) co-expression network topology within the **(B)** TM1_At (TA) and **(C)** H7124_At (HA) subgenome networks. Red and blue dashed lines designate the preservation thresholds at *Z*_summary_ = 10 and *Z*_summary_ = 2, respectively. According to standard criteria, *Z*_summary_ *>*10 signifies strong preservation, 2 ≤ *Z*_summary_ ≤ 10 represents weak-to-moderately preservation, and *Z*_summary_ < 2 indicates a lack of significant topological conservation.

We further used the consensus network to quantify the topological divergence among the three cotton species. Two complementary statistics were employed to evaluate network preservation at different organizational levels. The Eigengene Network Preservation parameter (*D*) was used to assess the global relationships among module eigengenes between two co-expression networks. Because module eigengenes represent the first principal component of each module, *D* reflects the similarity of higher-order network organization across datasets and is therefore particularly suitable for evaluating the overall conservation or divergence of entire co-expression network architectures between different genomes or subgenomes. The results revealed profound topological differentiation between diploid and tetraploid stress-responsive architectures (**Figure 4A**). Specifically, the SXY1_A_2_ network exhibited the highest preservation with TM1_A_t_ (*D_SA-TA_* = 0.87, *P* < 0.05), whereas its preservation with H7124_A_t_ was markedly lower (*D_SA-HA_* = 0.83, *P*< 0.05; *D_SA-HA_ < D_SA-TA_, P* < 0.05). Given the reduced resilience of *G. barbadense* (H7124) to cold, heat, and drought, we hypothesize that this lineage underwent more radical transcriptional rewiring during its evolution. In contrast, the Module Preservation statistic *Z_summary_* evaluates the preservation of the internal topology of individual modules by integrating multiple density- and connectivity-based preservation measures. Therefore, *Z_summary_* identifies which specific modules are highly conserved, moderately conserved, or not preserved across networks, providing a complementary assessment at the individual module level. Module Preservation Test confirmed that all modules within the SXY1_A_2_ network were either highly (*Z_summary_* > 10) or moderately (2 ≤ *Z_summary_* ≤ 10) preserved within the TM1_A_t_ network (**Figure 4B**). In contrast, the SXY1_A_2_ network module SA_M14 showed no evidence of preservation in H7124_A_t_, while 13 other modules exhibited only moderate preservation (**Figure 4C**). Because global network architecture and individual module topology represent different hierarchical properties of co-expression networks, the combined use of *D* and *Z*_summary_ provides a comprehensive evaluation of network conservation at both the global network level and the individual module level, which could not be achieved using either statistic alone. Collectively, these findings provide compelling topological evidence for deciphering subgenome-specific evolutionary dynamics in cotton stress resilience.

### 3.7 Core candidate genes negatively correlated with cold stress

**Figure 5:**
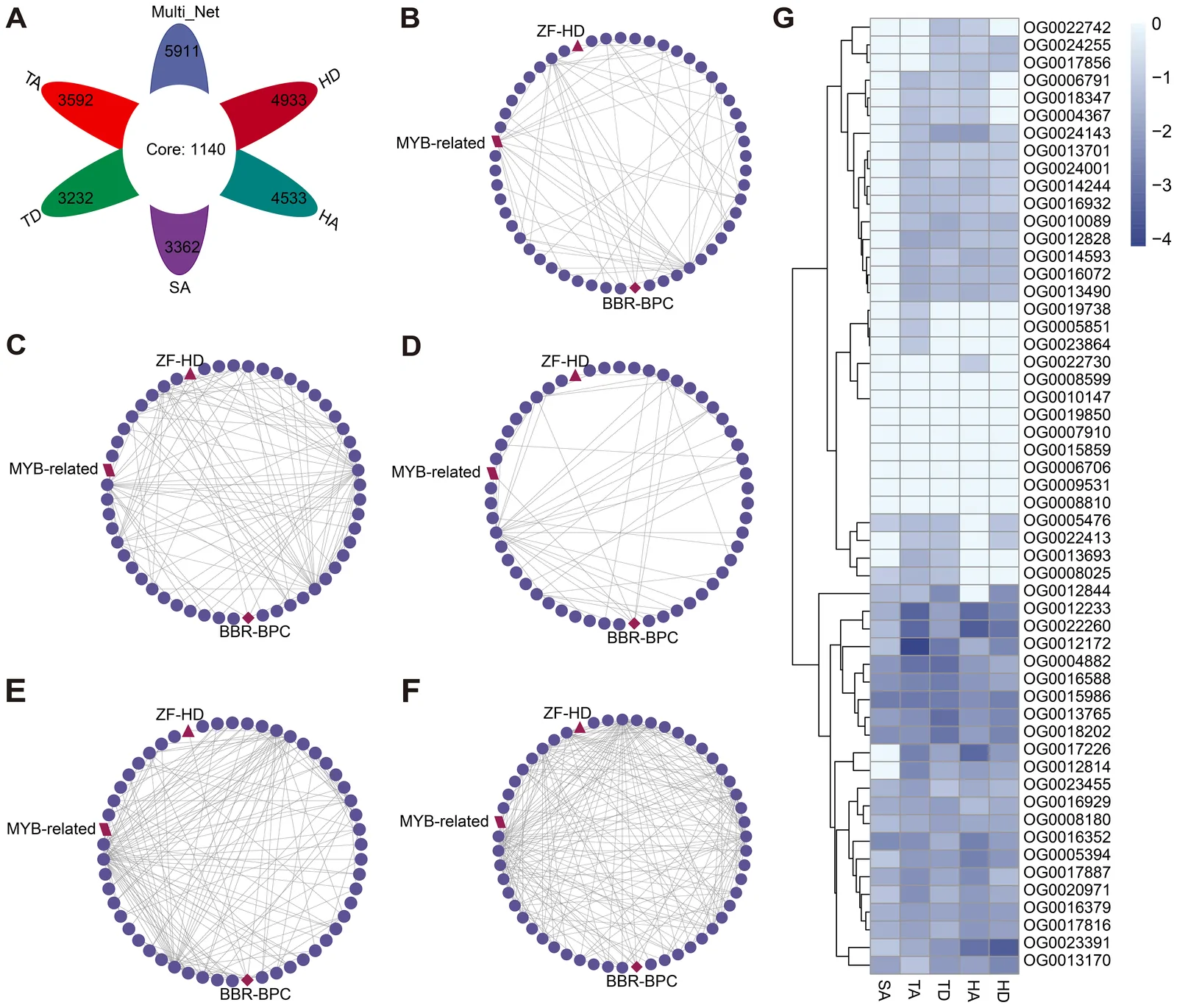
Identification and characterization of core gene sets significantly negatively correlated with cold stress. **(A)** Venn diagram illustrating the intersection of genes within Module 1 across the multi-species conserved network and the five constituent genomes networks: *G. arboreum* (SXY1_A_2_, SA), *G. hirsutum* (TM1_A_t_, TA), (TM1_D_t_, TD), and *G. barbadense* (H7124_A_t_, HA), (H7124_D_t_, HD). **(B–F)** Visualization of cold-responsive core sub-networks within the SA **(B)**, TA **(C)**, TD **(D)**, HA **(E)**, HD **(F)** co-expression frameworks (Pearson’s *r* > 0.95). Genes are arranged counterclockwise according to their genomic identifiers. **(G)** Differential expression profiles of the identified cold-responsive core genes. Heatmap color intensity represents the log_2_(fold-change) relative to the corresponding control (CK) groups.

Across the multi-species conserved stress network and the five independent genome networks, Module 1, the largest cluster by gene count, consistently displayed a significant negative correlation with cold stress (**Figure 3**; **Supplementary Figures 9**-**13**). By intersecting Module 1 across all genome networks, we identified a core assembly of 1,140 orthologous gene pairs (**Figure 5A**). Notably, 54 of the 59 cold-negative hub genes previously identified in the multi-species conserved network were included in this set, constituting a key candidate gene set for cotton cold-stress response. Despite high functional conservation, the intramodular co-expression topologies of this gene set exhibited pronounced divergence across disparate subgenomes (**Figures 5B–F**). Network analysis revealed substantial disparities in connectivity density and clustering coefficients among species (**Table 1**). Furthermore, differential expression profiling showed that these genes manifested heterogenous transcriptional patterns across the five genomic backgrounds (**Figure 5G**). Within this framework, three key transcription factors (TFs), belonging to the ZF-HD, BBR-BPC, and MYB-related families, remained prominent. Expression analysis indicated that the ZF-HD TF (*OG0016352*) underwent more drastic repression within the A-lineage (the diploid A-genome and two tetraploids A_t_ subgenome). In contrast, the BBR-BPC TF (*OG0004882*) exhibited more potent inhibitory responses in both subgenomes of TM1, whereas the MYB-related TF (*OG0018202*) showed a more significant downward trajectory in the two D_t_ subgenomes. Collectively, these results reveal that core cold-responsive genes in cotton have undergone functional divergence in expression magnitude during evolutionary selection.

**Table 1:** Comparative analysis of topological properties for the cold-responsive core sub-networks.

Networks	Density	Clustering coefficient
HD	0.6789^a^	0.6925^a^
HA	0.6293^b^	0.6467^b^
TA	0.5901^c^	0.6092^c^
TD	0.5816^c^	0.5968^d^
SA	0.5007^d^	0.5379^e^

Statistical significance is denoted by distinct lowercase letters (Kruskal–Wallis test followed by *post hoc* Dunn’s test with Benjamini-Hochberg adjusted, *P* < 0.05). SA: *G. arboreum*, SXY1_A_2_. TA and TD: *G. hirsutum*, TM1_A_t_ and TM1_D_t_. HA and HD: *G. barbadense*, H7124_A_t_ and H7124_D_t_.

### 3.8 Species-specific divergent modules in *G*. *arboreum*

**Figure 6:**
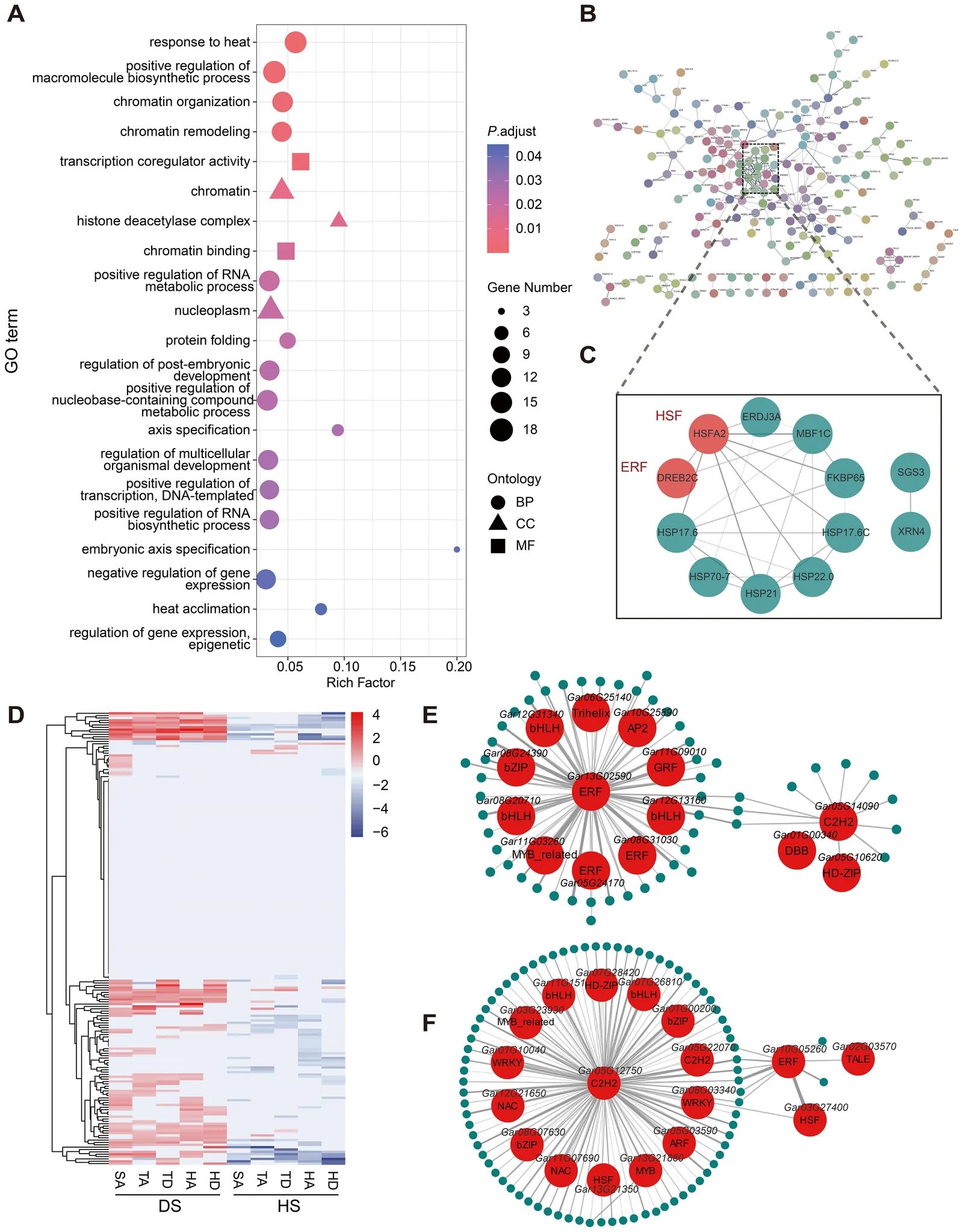
Functional dissection of the diverged modules SA_M14 and SA_M21 within the *G. arboreum* (A_2_) network. **(A)** Significant Gene Ontology (GO) terms enriched among *Arabidopsis thaliana* orthologs in module SA_M14 (Benjamini-Hochberg adjusted *P* < 0.05). **(B)** Protein–protein interaction (PPI) network architecture for module SA_M14. **(C)** Targeted sub-network within the PPI framework highlighting genes enriched in the “response to heat” category. **(D)** Differential expression profiles of genes within module SA_M21 across drought stress (DS) and heat stress (HS). **(E, F)** Reconstructed transcriptional regulatory networks for **(E)** module SA_M14 and **(F)** module SA_M21 (*P* < 0.05). Transcription factor (TF) nodes are highlighted in red and annotated with their respective family classifications.

Within the *G. arboreum* (SXY1_A_2_) network, we identified a specific module, SA_M14, which exhibited a lack of topological preservation in the H7124 network (**Figure 4C**; **Supplementary Table 3**). This module displayed a significant positive correlation with the heat stress response in *G. arboreum* (**Supplementary Figure 9**), suggesting its potential role in species-specific heat tolerance. GO enrichment analysis revealed that 17 genes within this module were significantly enriched in the “response to heat” term (GO:0009408, BH-adjusted *P* < 0.05) (**Figure 6A**). A protein-protein interaction (PPI) network, constructed based on *Arabidopsis* orthologs, delineated intricate associations among 12 heat-responsive proteins, 10 of which formed a highly interconnected core (**Figures 6B, C**). This interactome comprises five heat shock proteins (HSPs) along with HSF and ERF transcription factors. Notably, the *HSP17.6C* gene (*Gar09G05070*) and the *FKBP65* gene (*Gar10G16180*) were previously identified as hub genes within the heat-associated modules of the multi-species conserved network. Furthermore, TF regulatory network predictions placed the ERF TF (*Gar13G02590*) at the hierarchy’s core, displaying potential regulatory links with multiple downstream TFs (**Figure 6E**).

Additionally, we identified another diverged module in the SXY1_A_2_ network, SA_M21, characterized by an antagonistic correlation pattern: positive with drought but negative with heat stress (**Supplementary Figure 9**). This module showed moderately preservation across the remaining four subgenome networks (**Supplementary Table 3**). Differential expression profiling confirmed that genes within SA_M21 manifested diametrically opposed transcriptional trajectories under drought versus heat stress (**Figure 6D**). A regulatory network predicated on 36 intramodular TFs indicated that an ERF (*Gar10G05260*) potentially targets the promoter regions of the heat-responsive HSF (*Gar03G27400*) and the ABA-responsive TALE TF (*Gar02G03570*) (**Figure 6F**). These findings suggest that ethylene and ABA signaling pathways may coordinately contribute to the divergent responses of *G. arboreum* to drought and heat through specialized transcriptional circuits.

## 4 Discussion

### 4.1 A robust framework for cross-ploidy comparative transcriptomics

The establishment of a robust computational pipeline to mitigate interspecific heterogeneity is fundamental to cross-ploidy comparative transcriptomics. In this study, we achieved cross-genomic data integration by leveraging TPM values, with one to one orthologous/homoeologous gene pair identified in all subgenomes. To remove the confounding influence of genomic backgrounds during co-expression partitioning, we implemented a linear model treating genomic groups as batch-effect covariates, thereby successfully removing systematic variances. Validation confirmed that post-standardization data exhibited consistent distributions across subgenomes (**Figures 2C, D**, **Supplementary Figure 2**). This statistical rationale of eliminating evolutionary background noise via cross-species integration has been extensively implemented and validated in comparative transcriptomic studies of primate and rodent brains (Sjöstedt et al., 2020; Pembroke et al., 2021). This methodology provides a useful reference for comparative genomics, emphasizing the necessity of ensuring data scale consistency through logical partitioning, optimizing distributions via quantile normalization, and correcting for non-biological variables including sampling bias, technical platforms, and genomic heterogeneities.

While WGCNA is widely used for network inference, module stability is inherently sensitive to parameterization, such as soft-thresholding power and minimum module size, where stochastic fluctuations often cause substantial topological shifts. To bolster the robustness of network inference, strategies such as re-sampling (Monti et al., 2003) and bootstrapping, exemplified by the Similarity Across Bootstrap RE-sampling (SABRE) (Shannon et al., 2016) framework, have been proposed. The Bootstrapping Re-sampling Co-clustering Network Analysis (BRSCCNA) implemented herein, previously substantiated in diploid strawberry (Shahan et al., 2018) and kiwifruit (Zhang et al., 2022a), not only preserves correlation structures comparable to standard WGCNA but also significantly enhances the stability of gene membership through rigorous confidence interval assessments.

Although Consensus Analysis is traditionally deployed for multi-dataset integration (Morgan et al., 2020; Pembroke et al., 2021), precisely isolating conserved trait-associated modules across more than two datasets, such as the five genomic lineages examined here, presents formidable challenges. Consequently, we posit that for investigations involving three or more independent datasets, the “Standardization Pipeline + BRSCCNA Aggregated Network” strategy offers superior efficiency and fidelity in prioritizing target modules. Building upon this, we propose a streamlined comparative framework. Firstly, consolidating heterogeneous datasets via specialized normalization; secondly, identifying core modules and candidate gene cohorts through BRSCCNA aggregated networks; and finally, employing consensus network analysis to dissect lineage specific regulatory architectures. This workflow provides a robust technical foundation for investigating the molecular trajectories of phenotypic evolution and adaptive resilience during polyploidization. More broadly, this framework may be applicable to other polyploid crop systems, facilitating the dissection of regulatory diversification associated with the polyploidization.

### 4.2 Allopolyploidization driven remodeling of stress responsive co-expression networks

The phenotypic divergence between polyploids and their diploid progenitors, together with the evolution of their underlying regulatory frameworks, remains a cornerstone of evolutionary biology. While traditional paradigms posit that genetic redundancy derived from whole-genome duplication (WGD) confers enhanced environmental plasticity, recent investigations into salinity tolerance in cotton have challenged this “polyploid superiority” model. Indeed, allotetraploids do not universally outperform their diploid ancestors due to substantial intraspecific variation across disparate genomic groups (Dong et al., 2020), underscoring the nuanced impact of polyploidy on stress resilience. Our findings further corroborate this phenomenon: the diploid *G. arboreum* manifests superior heat tolerance and drought resistance, whereas *G. hirsutum* excels in cold tolerance, while *G. barbadense* exhibits relatively diminished resilience across cold, heat, and drought stresses.

Network topological analysis revealed pronounced divergence in stress-responsive architectures between diploid and tetraploid cotton. Specifically, the inter-module connectivity within the *G. barbadense* (H7124_A_t_/D_t_) subgenomes networks was the highest, followed by *G. hirsutum* (TM1_A_t_/D_t_), while *G. arboreum* (SXY1_A_2_) exhibited the lowest connectivity (**Figures 4A**, **5**). This heightened connectivity in *G. barbadense* may reflect “regulatory entanglement” or non-functional redundancy, potentially sequestering the regulatory plasticity required to navigate specific environmental adversities. Analogous to the extensive remodeling of seed oil metabolic networks previously documented during allopolyploidization (Hu et al., 2016), our findings suggest that the observed network divergence is consistent with transcriptional remodeling accompanying polyploid evolution.

Within the A-genome lineage, the stress networks of *G. hirsutum* A_t_ and *G. arboreum* A_2_ maintain robust conservation. Conversely, *G. barbadense* A_t_ exhibited substantial divergence in stress-responsive network topology, which may be associated with its relatively reduced stress resilience. Furthermore, our results indicate that the network architectures between the A_t_ and D_t_ subgenomes within both *G. hirsutum* and *G. barbadense* are highly conserved. This observation aligns with previous findings in *G. hirsutum* fiber co-expression networks, suggesting that co-existing subgenomes tend to maintain structural network conservation (Gallagher et al., 2020).

Extensive transcriptional rewiring in the *G. barbadense* stress-responsive network may, at least in part, reflect the cumulative effects of homoeolog expression bias that accompanies allopolyploid evolution. Previous studies have demonstrated widespread subgenome-biased expression in allotetraploid cotton, providing a potential mechanism by which regulatory interactions can diverge despite the conservation of gene content. Although homoeolog expression bias was not explicitly quantified in the present study, the pronounced topological divergence observed in *G. barbadense* is consistent with long-term regulatory remodeling following polyploidization.

Moreover, *G. barbadense* has generally been regarded as possessing a relatively narrower ecological adaptability than *G. hirsutum* despite its superior fiber quality. It is therefore conceivable that evolutionary selection favoring fiber-related traits may have been accompanied by reduced conservation of stress-responsive regulatory networks. This interpretation should be regarded as a biologically plausible hypothesis requiring further investigation.

Notably, the extensive erosion of network topology coincided with the relatively weaker tolerance of *G. barbadense* to cold, heat, and drought stresses observed in this study. We therefore propose that transcriptional rewiring may weaken the coordinated regulation of conserved stress-response pathways, thereby contributing to the reduced stress resilience of this lineage. However, because co-expression networks identify regulatory associations rather than causal relationships, experimental validation will be required to establish the mechanistic contribution of network rewiring to stress adaptation.

## 5 Conclusions

In summary, this study systematically characterized the divergent abiotic stress tolerance profiles of three representative cotton species. By establishing a dedicated cross-ploidy transcriptomic standardization pipeline, we enabled systematic comparison of conserved and divergent abiotic stress-responsive gene co-expression networks across diploid and allotetraploid cotton genomes. Integrating multi-species co-expression analysis with genome-specific network analyses allowed us to prioritize conserved modules, lineage-specific modules, and candidate regulatory genes potentially associated with cotton stress adaptation. Collectively, our findings provide a comparative framework for investigating the evolutionary diversification of stress-responsive regulatory networks during cotton polyploidization and offer candidate genes and analytical strategies that may facilitate future functional studies and the molecular improvement of stress-resilient cotton cultivars.

## Data Availability

The datasets presented in this study can be found in online repositories. The names of the repository/repositories and accession number(s) can be found below: https://www.ncbi.nlm.nih.gov/, PRJNA631851, PRJNA1463507, PRJNA1463331.
