# Supplementary material for: Conservation and divergence of abiotic stress-responsive gene co-expression networks in diploid and tetraploid cotton
Source: Front Plant Sci. 2026 Aug 12;17:1889274. doi: 10.3389/fpls.2026.1889274 (PMC13506909; doi:10.3389/fpls.2026.1889274)
Supplement: Supplementary file 1 [file SupplementaryFile1.docx]

Supplementary Material

**Conservation and divergence of abiotic stress-responsive gene co-expression networks in diploid and tetraploid cotton**

**Heng Wang^1^, Yumeng Zhu^1^, Ting Zhao^1^, Xueying Guan^1^, Junduo Wang^2*^, and Luyao Wang^1,3*^**

^1^Zhejiang Provincial Key Laboratory of Crop Genetic Resources, Institute of Crop Science, Plant Precision Breeding Academy, College of Agriculture and Biotechnology, Zhejiang University, Hangzhou, China, ^2^Xinjiang Key Laboratory of Cotton Genetic Improvement and Intelligent Production/Xinjiang Cotton Technology Innovation Center, Cotton Research Institute of Academy of Agricultural Sciences of Xinjiang Uyghur Autonomous Region (National Cotton Engineering Technology Research Center), Urumqi, China, ^3^Tropical Crops Genetic Resources Institute, Chinese Academy of Tropical Agricultural Sciences/Key Laboratory of Crop Gene Resources and Germplasm Enhancement in Southern China, Ministry of Agriculture and Rual Affairs/Key Laboratory of Tropical Crops Germplasm Resources Genetic Improvement and Innovation of Hainan Province, Haikou, China

**^*^Correspondence:**

Junduo Wang

13579975299@163.com

Luyao Wang

[2171100066@zju.edu.cn](mailto:2171100066@zju.edu.cn)

**Supplementary Figure 1:** Assessment of transcriptomic reproducibility across RNA-seq libraries.

**Supplementary Figure 2:** Statistical distribution of expression profiles for orthologous and homoeologous gene pairs across five subgenomes.

**Supplementary Figure 3:** Hierarchical clustering and inter-module correlations of eigengenes within the multi-species co-expression network.

**Supplementary Figure 4:** Intramodular linear regression analysis between module membership (kME) and gene significance (GS) across the multi-species conserved co-expression network.

**Supplementary Figure 5:** Transcriptional profiles of module eigengenes strongly associated with abiotic stressors.

**Supplementary Figure 6:** Functional enrichment analysis of stress-responsive co-expression modules.

**Supplementary Figure 7:** Global conservation of module–trait correlations across five cotton subgenomes.

**Supplementary Figure 8:** Overlap significance between consensus modules and multi-species conserved network modules.

**Supplementary Figure 9:** Correlations between *G. arboreum* (A_2_) co-expression modules and abiotic stressors.

**Supplementary Figure 10:** Correlation between *G. hirsutum* (A_t_) subgenomic co-expression modules and abiotic stressors.

**Supplementary Figure 11:** Correlation between *G. hirsutum* (D_t_) subgenomic co-expression modules and abiotic stressors.

**Supplementary Figure 12:** Correlation between *G. barbadense* (A_t_) subgenomic co-expression modules and abiotic stressors.

**Supplementary Figure 13:** Correlation between *G. barbadense* (D_t_) subgenomic co-expression modules and abiotic stressors.

**Supplementary Table 1:** Summary of RNA-seq data.

**Supplementary Table 2:** Annotations of hub genes from modules significantly related with abiotic stresses in the multi-species co-expression network.

**Supplementary Table 3:** Module preservation of *G. arboreum* (SXY1_A_2_) co-expression network.

**Supplementary Table 4:** Annotations of module SA_M14 within the *G. arboreum* (A_2_) co-expression network.

**Supplementary Table 5:** Annotations of module SA_M21 within the *G. arboreum* (A_2_) co-expression network.

**Supplementary** **Figure 1. Assessment of transcriptomic reproducibility across RNA-seq libraries.**

(**A-C**) Heatmaps illustrating Pearson correlation coefficients among biological replicates and experimental treatments for *G. hirsutum* (TM1), *G. barbadense* (H7124), and *G. arboreum* (SXY1). The color scale represents the strength of the linear relationship, with coefficients approaching 1.0 indicating high transcriptional consistency between samples.

**Supplementary** **Figure 2. Statistical distribution of expression profiles for orthologous and homoeologous gene pairs across five subgenomes.**

Violin plots illustrating the density distribution of transcript abundance at different stages of normalization: (**A**) initial (log_2_(TPM+1)) values; (**B**) expression levels after quantile normalization (Normalized expression 1, as delineated in Fig. 2B); and (**C**) expression levels following the removal of species-specific genome background effects (Normalized expression 2, as delineated in Fig. 2B). Data are presented for two control (CK) groups and four abiotic stress groups across *G. hirsutum* (TM1_A_t_/D_t_), *G. barbadense* (H7124_A_t_/D_t_), and *G. arboreum* (SXY1_A_2_).

**Supplementary** **Figure 3. Hierarchical clustering and inter-module correlations of eigengenes within the multi-species co-expression network.**

(**A**) Hierarchical clustering dendrogram illustrating the topological relationships among module eigengenes. (**B**) Heatmap depicting Pearson correlation coefficients between respective module eigengenes, revealing the transcriptional co-regulation or independence among identified co-expression modules.

**Supplementary** **Figure 4. Intramodular linear regression analysis between module membership (kME) and gene significance (GS) across the multi-species conserved co-expression network.**

(**A-I**) Scatter plots illustrating the correlation between kME and GS for key stress-responsive modules: M3, M1, M14, M32, M6, M19, M4, M26, and M33, respectively. The y-axis in each panel represents the gene significance associated with a specific abiotic stressor: (**A-B**) cold stress (CS), (**C-E**) drought stress (DS), (**F-G**) heat stress (HS), and (**H-I**) salinity stress (SS). High correlation coefficients and statistical significance within these modules validate the robustness of the identified stress-responsive regulatory hubs.

**Supplementary** **Figure 5. Transcriptional profiles of module eigengenes strongly associated with abiotic stressors.**

(**A-I**) Expression dynamics of eigengenes for key stress-responsive modules: M3, M1, M14, M32, M6, M19, M4, M26, and M33, respectively. Bar plots illustrate eigengene values, mean-centered across all genomic backgrounds and stress cohorts to highlight relative expression shifts. Error bars denote the standard error (SE) derived from three biological replicates per genome per treatment. Module identifiers, their assigned colors, and the total number of constituent genes are indicated above each respective panel.

**Supplementary** **Figure 6. Functional enrichment analysis of stress-responsive co-expression modules.**

Dot plots illustrate the Gene Ontology (GO) enrichment results for modules M3 (**A**), M1 (**B**), M6 (**C**), and M19 (**D**). The y-axis displays the top 20 significantly enriched GO terms, prioritized by their Benjamini-Hochberg (BH) adjusted *P*-values (*P* < 0.05). Dot size is proportional to the number of genes assigned to each term, while the color gradient represents the magnitude of statistical significance based on the BH-adjusted *P*-value.

**Supplementary** **Figure 7. Global conservation of module–trait correlations across five cotton subgenomes.**

Consensus module-stress correlation frameworks were established by integrating five discrete genomic datasets: *G. arboreum* (SXY1_A_2_), *G. hirsutum* (TM1_A_t_, D_t_), and *G. barbadense* (H7124_A_t_, D_t_). For each module-stress pair, the consensus correlation was derived using a highly conservative filtering logic: a non-zero correlation was retained only if the coefficients displayed concordant polarities across all five datasets, in which case the minimum absolute value among them was assigned; conversely, the relationship was designated as zero if any discordant polarities (opposite signs) were detected. Module eigengenes (rows, designated by module ID and assigned color) were correlated with specific abiotic stress cohorts (columns). CK denotes the control group associated with cold stress (CS), while CK_2 serves as the unified control for drought stress (DS), heat stress (HS), and salinity stress (SS). Heatmap colors represent the consensus Pearson correlation coefficients, and asterisks indicate Benjamini-Hochberg (BH) adjusted *P*-values (**P* < 0.05; ** *P* <0.01; *** *P* <0.001).

**Supplementary** **Figure 8. Overlap significance between consensus modules and multi-species conserved network modules.**

The correspondence between modules identified via consensus analysis and those from the multi-species conserved network was evaluated using Fisher’s exact test. The color gradient in the heatmap represents the significance of gene overlap, expressed as -log_10_(*P*-value). High values (red) denote substantial and statistically significant modular congruency between the two independent network construction methodologies.

**Supplementary** **Figure 9. Correlations between *G. arboreum* (A_2_) co-expression modules and abiotic stressors.**

SA: *G. arboreum* SXY1_A_2_. Module eigengenes (rows, designated by module ID and assigned color) were correlated with specific abiotic stress cohorts (columns). CK denotes the control group associated with cold stress (CS), while CK_2 serves as the unified control for drought stress (DS), heat stress (HS), and salinity stress (SS). The heatmap color scale represents Pearson correlation coefficients, and asterisks indicate Benjamini-Hochberg (BH) adjusted *P*-values (**P* < 0.05; ** *P* <0.01; ****P* <0.001).

**Supplementary** **Figure 10. Correlation between *G. hirsutum* (A_t_) subgenomic co-expression modules and abiotic stressors.**

TA: *G. hirsutum* TM1_A_t_. Module eigengenes (rows, designated by module ID and assigned color) were correlated with specific abiotic stress cohorts (columns). CK denotes the control group associated with cold stress (CS), while CK_2 serves as the unified control for drought stress (DS), heat stress (HS), and salinity stress (SS). The heatmap color scale represents Pearson correlation coefficients, and asterisks indicate Benjamini-Hochberg (BH) adjusted *P*-values (**P* < 0.05; ***P* <0.01; *** *P* <0.001).

**Supplementary** **Figure 11. Correlation between *G. hirsutum* (D_t_) subgenomic co-expression modules and abiotic stressors.**

TD: *G. hirsutum* TM1_D_t_. Module eigengenes (rows, designated by module ID and assigned color) were correlated with specific abiotic stress cohorts (columns). CK denotes the control group associated with cold stress (CS), while CK_2 serves as the unified control for drought stress (DS), heat stress (HS), and salinity stress (SS). The heatmap color scale represents Pearson correlation coefficients, and asterisks indicate Benjamini-Hochberg (BH) adjusted *P*-values (**P* < 0.05; ***P* <0.01; ****P* <0.001).

**Supplementary** **Figure 12. Correlation between *G. barbadense* (A_t_) subgenomic co-expression modules and abiotic stressors.**

HA: *G. barbadense* H7124_A_t_. Module eigengenes (rows, designated by module ID and assigned color) were correlated with specific abiotic stress cohorts (columns). CK denotes the control group associated with cold stress (CS), while CK_2 serves as the unified control for drought stress (DS), heat stress (HS), and salinity stress (SS). The heatmap color scale represents Pearson correlation coefficients, and asterisks indicate Benjamini-Hochberg (BH) adjusted *P*-values (**P* < 0.05; ***P* <0.01; ****P* <0.001).

**Supplementary** **Figure 13. Correlation between *G. barbadense* (D_t_) subgenomic co-expression modules and abiotic stressors.**

HD: *G. barbadense* H7124_D_t_. Module eigengenes (rows, designated by module ID and assigned color) were correlated with specific abiotic stress cohorts (columns). CK denotes the control group associated with cold stress (CS), while CK_2 serves as the unified control for drought stress (DS), heat stress (HS), and salinity stress (SS). The heatmap color scale represents Pearson correlation coefficients, and asterisks indicate Benjamini-Hochberg (BH) adjusted *P*-values (**P* < 0.05; ***P* <0.01; ****P* <0.001).
